# 62. Follow-Up Blood Culture Practices for Gram-Negative Bloodstream Infections in Immunocompromised Hosts at a Large Academic Medical Center

**DOI:** 10.1093/ofid/ofab466.062

**Published:** 2021-12-04

**Authors:** Lauren Groft, James Mease, Jacqueline Bork, Ciera L Bernhardi, J Kristie Johnson, Kimberly C Claeys

**Affiliations:** 1 The Johns Hopkins Hospital, Baltimore, MD; 2 University of Maryland School of Pharmacy, Baltimore, Maryland; 3 University of Maryland School of Medicine, Baltimore, Maryland; 4 University of Maryland Medical Center, Arnold, Maryland; 5 University of Maryland, Baltimore, MD

## Abstract

**Background:**

Routine follow-up blood cultures (FUBC) are strongly recommended for *Staphylococcus aureus* and *Candida* spp. bloodstream infections (BSI), but the role of FUBC in Gram-negative (GN) BSI remains controversial. Factors that may result in persistent GN BSI include critical illness, endovascular infection, lack of source control, multidrug resistant organisms (MDRO), end-stage renal disease, or immunocompromised status. As such, FUBC in patients with any of these factors may be warranted to improve clinical outcomes, but the true balance of benefit versus harm remains unknown. Our objective was to evaluate the role of FUBC in immunocompromised patients with GN BSI.

**Methods:**

This was a retrospective observational cohort of adult, immunocompromised patients treated for confirmed GN BSI between January 2019 and December 2020 at University of Maryland Medical Center. Immunocompromise was defined as active hematologic or solid tumor malignancy at time of BSI diagnosis, history of hematopoietic stem cell transplantation (HSCT) or solid organ transplantation (SOT), or absolute neutrophil count (ANC) < 1000 cells/mm^3^ at any time 30 days prior to BSI diagnosis. FUBC were defined as blood cultures drawn between 24 hours and 7 days from index blood culture, within the same hospital encounter. Positive FUBC was defined as a FUBC with same pathogenic GN organism identified. Comparison of patient and microbiologic characteristics was made between patients with and without FUBC.

**Results:**

A total of 146 patients with GN BSI were included. Baseline characteristics are reported in Table 1. FUBC were collected in 129 (88.4%) patients. Neutropenia (49.6% vs. 19.4%, *P*=0.122), presence of central line (69.8% vs. 30.2%, *P*=0.061), and hospital-acquired origin of BSI (63.6% vs. 36.4%, *P*=0.395) resulted in increased frequency of FUBC. Patients with FUBC had a significantly longer post-BSI mean (SD) length of stay (17.3 [35.4] vs. 6.5 [6.0] days; *P*=0.005). Positive FUBC occurred in only 2 cases (1.4%) and both patients had persistent fevers at time of FUBC.

Table 1. Baseline Characteristics

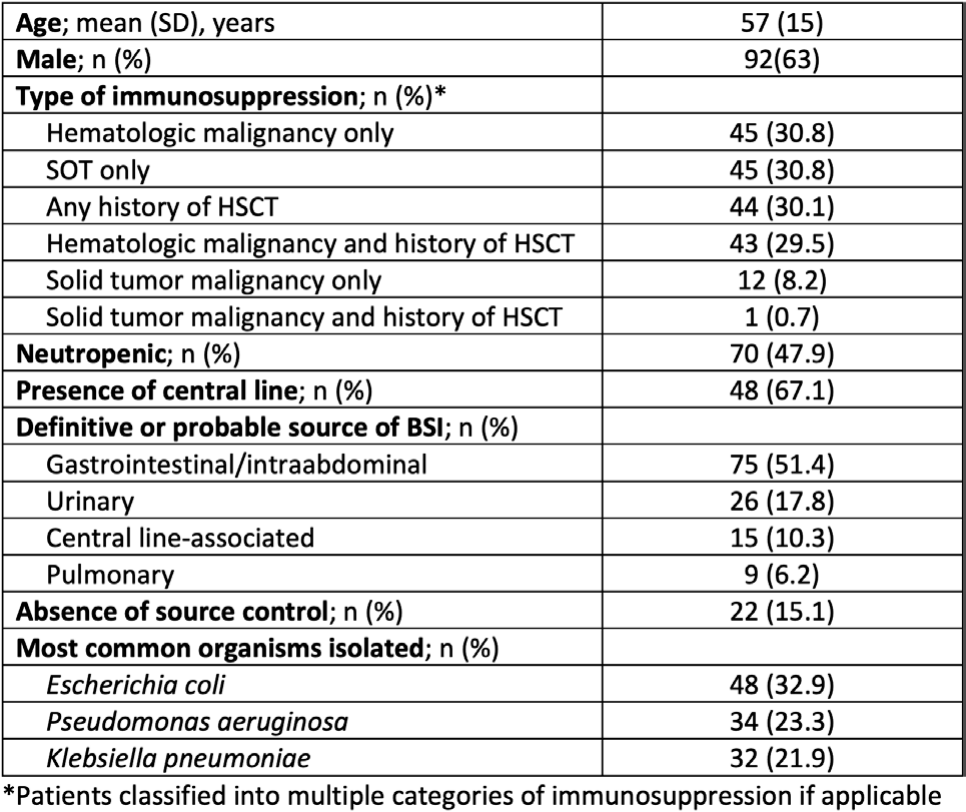

**Conclusion:**

Positive FUBC were uncommon in this immunocompromised cohort with GN BSI, which challenges the need for routine collection of FUBC in this patient population.

**Disclosures:**

**Ciera L. Bernhardi, PharmD**, **Servier Pharmaceuticals** (Advisor or Review Panel member) **J. Kristie Johnson, PhD, D(ABMM**), **GenMark** (Speaker’s Bureau) **Kimberly C. Claeys, PharmD**, **GenMark** (Speaker’s Bureau)

